# Supplementary material for: Brief Magnetic Field Exposure Stimulates Doxorubicin Uptake into Breast Cancer Cells in Association with TRPC1 Expression: A Precision Oncology Methodology to Enhance Chemotherapeutic Outcome
Source: Cancers (Basel). 2024 Nov 18;16(22):3860. doi: 10.3390/cancers16223860 (PMC11592624; doi:10.3390/cancers16223860)
Supplement: Supplementary file 1 [file cancers-16-03860-s001.zip › cancers-3296954-Figures S1 and S2.pdf]

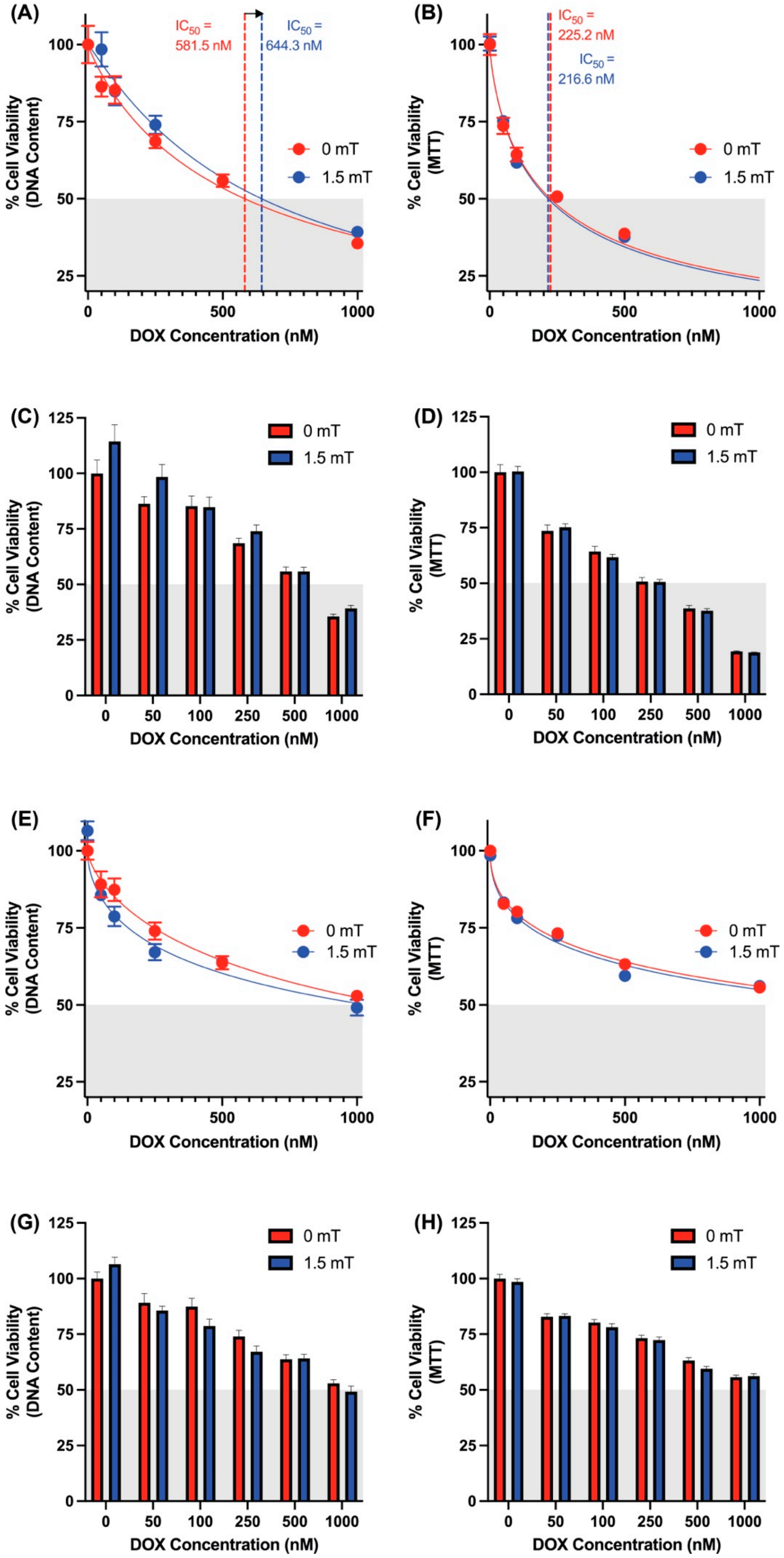

(A)
IC50 =
581.5 nM
IC50 =
644.3 nM
% Cell Viability (DNA Content)
0 mT
1.5 mT
DOX Concentration (nM)
(B)
IC50 =
225.2 nM
IC50 =
216.6 nM
% Cell Viability (MTT)
0 mT
1.5 mT
DOX Concentration (nM)
(C)
% Cell Viability (DNA Content)
0 mT
1.5 mT
DOX Concentration (nM)
(D)
% Cell Viability (MTT)
0 mT
1.5 mT
DOX Concentration (nM)
(E)
% Cell Viability (DNA Content)
0 mT
1.5 mT
DOX Concentration (nM)
(F)
% Cell Viability (MTT)
0 mT
1.5 mT
DOX Concentration (nM)
(G)
% Cell Viability (DNA Content)
0 mT
1.5 mT
DOX Concentration (nM)
(H)
% Cell Viability (MTT)
0 mT
1.5 mT
DOX Concentration (nM)

Figure S1. Muscle cells are not susceptible to PEMF-enhanced, DOX-mediated, cytotoxicity. **(A)** Dose-response curve of C2C12 myoblasts treated with increasing concentrations of DOX for 24 h, with IC_{50} values as extrapolated from the red (unexposed, IC_{50} = 581.5 nM or blue (muscle signature - magnetically exposed, IC_{50} = 644.3 nM) symbols as generated using cellular DNA content. **(B)** Dose-response curve of C2C12 myoblast cells treated with increasing concentrations of DOX for 48 h. IC_{50} values as extrapolated from the red (unexposed), or blue (muscle signature - magnetically exposed) symbols as generated measuring mitochondrial respiration (MTT). **(C)** Bar chart corresponding to panel **A** showing % cell viability of C2C12 myoblasts treated with increasing concentrations of DOX for 24 h after treatment with magnetic fields and DOX. **(D)** Bar chart corresponding to panel **B** showing % cell viability of C2C12 myoblasts treated with increasing concentrations of DOX for 48 h after treatment with magnetic fields and DOX. **(E)** Dose-response curve of C2C12 myotube cells treated with increasing concentrations of DOX for 24 h. **(F)** Dose-response curve of C2C12 myotube cells treated with increasing concentrations of DOX for 48 h. **(G)** Bar chart corresponding to panel **E** showing % cell viability of C2C12 myotubes treated with increasing concentrations of DOX for 24 h after treatment with magnetic fields and DOX. **(H)** Bar chart corresponding to panel **F** showing % cell viability of C2C12 myotubes treated with increasing concentrations of DOX for 48 h after treatment with magnetic fields and DOX. Panels (**A**,**C**,**E**,**G**) were generated using a CyQuant DNA content assay kit as described in **Section 2.** Panels (**B**,**D**,**F**,**H**) were generated using a MTT assay kit as described in **Section 2.** In all cases, DOX was added 5 min prior to magnetic stimulation for 10 min. Data represent mean ± standard error of the mean (SEM) (n = 4, with 8 technical replicates for myoblasts, 4 technical replicates for myotubes). Statistical analysis was performed using 2-way ANOVA, followed by Šidák's multiple comparison post hoc test.

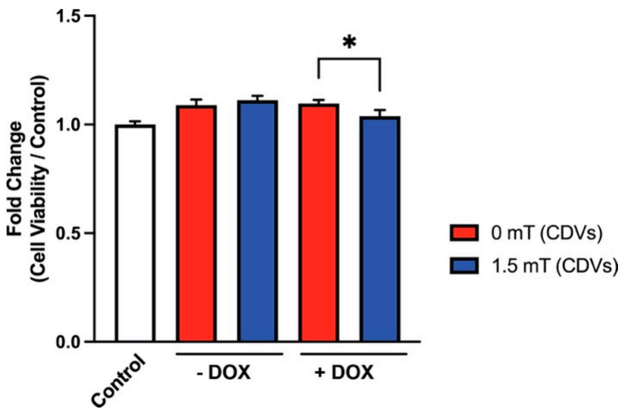

Figure S2. Magnetic exposure loads CDVs with DOX that can be then transferred to breast cancer cells for targeted killing. Fold change in the viability of MDA-MB231 human breast cancer cells after treatment with CDVs that were either magnetically exposed (1.5 mT), or not (0 mT) for 10 min, while in the absence or presence of 100 nM DOX. Cell viability was ascertained by quantifying cellular DNA using CyQuant as described in **Section 2.** Each repetition of a condition was provisioned with the quantity of CDVs generated from approximately 1.5 x 10^6 wild type C2C12 myoblasts. An optimization of the concentration of CDVs to observe the greatest cell response was not attempted here; the only objective was to detect differential cell responses. Data represent mean ± standard error of the mean (SEM) (n = 3, with 6 technical replicates). Statistical analysis was performed using Student's *t*-test. Significance is indicated by *, $p \leq 0.05$.
