# Supplementary figures and images for: Brief Magnetic Field Exposure Stimulates Doxorubicin Uptake into Breast Cancer Cells in Association with TRPC1 Expression: A Precision Oncology Methodology to Enhance Chemotherapeutic Outcome
Source: Cancers (Basel). 2024 Nov 18;16(22):3860. doi: 10.3390/cancers16223860 (PMC11592624; doi:10.3390/cancers16223860)

Figure 3E Western Blot of GFP-TRPC1 in MCF7 and MCF7-TRPC1 cells

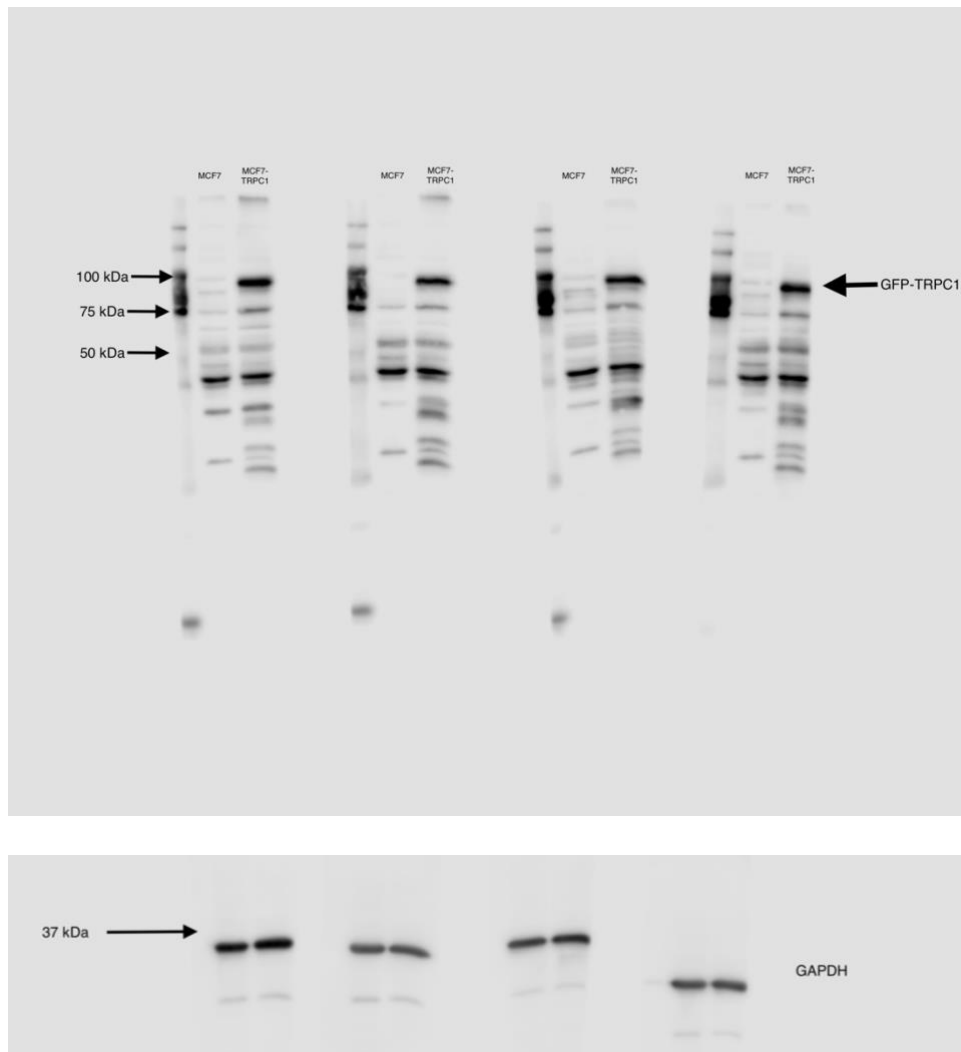

Supplement: Supplementary file 1 [file cancers-16-03860-s001.zip › cancers-3296954-Figure S3.pdf]
